# Continuous Estimation of Blood Pressure by Utilizing Seismocardiogram Signal Features in Relation to Electrocardiogram

**DOI:** 10.3390/bios14120621

**Published:** 2024-12-17

**Authors:** Aleksandra Zienkiewicz, Vesa Korhonen, Vesa Kiviniemi, Teemu Myllylä

**Affiliations:** 1 Optoelectronics and Measurement Techniques Research Unit, University of Oulu, 90570 Oulu, Finland; 2 Oulu Functional Neuroimaging, Department of Diagnostic Radiology, Oulu University Hospital, 90220 Oulu, Finland; 3 Research Unit of Health Sciences and Technology, University of Oulu, 90220 Oulu, Finland

**Keywords:** blood pressure, pulse transit time, pulse arrival time, seismocardiogram

## Abstract

There is an ongoing search for a reliable and continuous method of noninvasive blood pressure (BP) tracking. In this study, we investigate the feasibility of utilizing seismocardiogram (SCG) signals, i.e., chest motion caused by cardiac activity, for this purpose. This research is novel in examining the temporal relationship between the SCG-measured isovolumic moment and the electrocardiogram (PEP_IM_). Additionally, we compare these results with the traditionally measured pre-ejection period with the aortic opening marked as an endpoint (PEP_AO_). The accuracy of the BP estimation was evaluated beat to beat against invasively measured arterial BP. Data were collected on separate days as eighteen sets from nine subjects undergoing a medical procedure with anesthesia. Results for PEP_IM_ showed a correlation of 0.67 ± 0.18 (*p* < 0.001), 0.66 ± 0.17 (*p* < 0.001), and 0.67 ± 0.17 (*p* < 0.001) when compared to systolic BP, diastolic BP, and mean arterial pressure (MAP), respectively. Corresponding results for PEP_AO_ were equal to 0.61 ± 0.22 (*p* < 0.001), 0.61 ± 0.21 (*p* < 0.001), and 0.62 ± 0.22 (*p* < 0.001). Values of PEP_IM_ were used to estimate MAP using two first-degree models, the linear regression model (achieved RMSE of 11.7 ± 4.0 mmHg) and extended model with HR (RMSE of 10.8 ± 4.2 mmHg), and two corresponding second-degree models (RMSE of 10.8 ± 3.7 mmHg and RMSE of 8.5 ± 3.4 mmHg for second-degree polynomial and second-degree extended, respectively). In the intrasubject testing of the second-degree model extended with HR based on PEP_IM_ values, the mean error of MAP estimation in three follow-up measurements was in the range of 7.5 to 10.5 mmHg, without recalibration. This study demonstrates the method’s potential for further research, particularly given that both proximal and distal pulses are measured in close proximity to the heart and cardiac output. This positioning may enhance the method’s capacity to more accurately reflect central blood pressure compared to peripheral measurements.

## 1. Introduction

The rhythmic motion of the chest, resulting from intricate cardiac dynamics, has been of long-standing interest to researchers, as it can provide a noninvasive glimpse into the cardiovascular health of an individual and be complementary to methods focusing on the heart’s electrical activity. Measurement of the miniscule vibrations, caused by mechanical activity of the heart together with the surrounding tissue and body liquids, is called seismocardiography (SCG). It is obtained using an accelerometer, and although all three orthogonal body axes can provide unique responses, most SCG research primarily examines the changes in the dorsoventral component [1]. SCG has an established position in cardiac studies, especially for tracking heart and breathing rates [1,2,3,4], and is suggested for other applications such as the estimation of stroke volume [5], heart failure clinical status [6,7], or cardiorespiratory fitness [8]. However, surprisingly little has been achieved in terms of utilizing the technique in continuous noninvasive blood pressure (BP) estimation.

Since the most used noninvasive BP measurement method is still an intermittent measurement with a bulky brachial cuff, there is an ongoing search for accurate alternatives. One of the distinct trends in the development of alternative techniques for BP estimation is measurement of pulse arrival time (PAT), which is the time duration of a pulse wave propagating from the proximal artery to the distal location, with the proximal timing being measured using an electrocardiogram (ECG). Distal timing is measured at a peripheral site on the body, e.g., the finger, ear, toe, or forehead. However, the pressure wave propagation can also be measured using other sensors and sensor placements [9,10]. Despite being tested in numerous studies, there is no consensus on which measurement site provides the best outcomes, and the reproducibility of the results is often challenging [11,12]. PAT also includes the pre-ejection period, PEP: the time delay between the electrical depolarization of the heart’s left ventricle (ECG R-peak) and the ejection of blood through the aorta. PEP is a non-constant delay, which changes rapidly in response to stress, emotion, and physical effort; it also may change due to heart dysfunction and increases with age [13]. Within the literature, there exist some inconsistencies with regard to the relation between PEP and BP, as in some studies, it has been reported to be correlated with systolic BP [14,15], while in other studies, the influence of PEP on BP estimation was considered detrimental [16,17]. Since it is the accurate time definition that is crucial in PEP measurement, utilizing the measurement of heart mechanic activity has a justified place.

**Figure 1:**
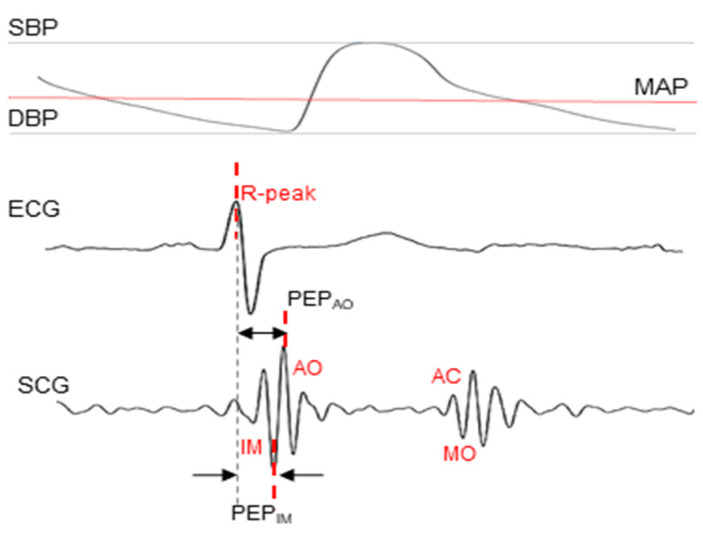
Characteristic points of simultaneously detected signals: invasive BP, ECG, and SCG. IM: isovolumic movement; AO: aortic value opening; AC: aortic valve closure; MO: mitral valve opening.

Throughout the cardiac cycle, variations in contraction forces produce distinct patterns in the SCG signal, which can be observed with similar replicability to ECG waves. Peaks and valleys known as fiducial points represent specific events in the heart, such as mitral valve closure (MC) and aortic valve opening (AO) peaks, related to systolic activity, and aortic valve closure (AC) and mitral valve opening (MO) peaks, during the diastolic phase (see Figure 1). The relationship between these points and phases of cardiac activity was defined by simultaneous measurement with ECG and an echocardiogram [18]; however, the interpretation of fiducial points might still differ between studies and measurement techniques [19,20,21]. Peaks such as AO are fairly straightforward results of motion during aortic valve opening; its interpretation as the highest maximum peak in an SCG systolic pulse is relatively consistent between studies. AO in combination with the ECG R-peak is widely used as a way to measure PEP [22]. However, points such as an isovolumic moment (IM), which is a prominent minimum peak right before AO, appear to hold a distinct significance solely within the context of the SCG waveform [23], as IM is defined as the minimum peak of the SCG signal during isovolumic contraction (starting with the closure of the mitral valve and ending with the opening of the aortic valve).

In the presented method, we propose to use the ECG R-peak and SCG IM point for measurement of electro-mechanical delay and use it for BP estimation. We measured SCG by placing on the chest a one-dimensional accelerometer, which senses dorsoventral axis accelerations. We detected AO and IM points in SCG pulses, and by calculating the delay from the simultaneously measured ECG R-peak, we obtained PEP_AO_ and PEP_IM_, respectively. PEP_AO_ directly corresponds to PEP as defined in the literature, whereas PEP_IM_ is dominated by the heart’s isovolumic moment [24,25]. The abbreviation PEP_IM_ was chosen as it also appears before blood ejection during aortic valve opening. Determined PEP was compared to invasively measured BP (IBP) and used as a base for BP estimation using linear and polynomial regression models. To the best of our knowledge, it is the first time for the SCG IM point to be used in BP estimation.

## 2. Materials and Methods

In this study, eighteen datasets were measured, each on different days, from nine subjects (mean age ± SD = 56 ± 16 years, range = 20–68, 5 females; see Table 1). Written informed consent was obtained from each patient prior to the procedure in addition to routine clinical treatment information. The study was carried out in accordance with the Declaration of Helsinki and approved by the Ethical Committee of the Northern Ostrobothnia Hospital District, Oulu University Hospital (number 5/2014).

Data were collected from patients in the supine position, during, i.a., mannitol treatment for blood–brain barrier (BBB) opening to augment by 10–100-fold the entry of chemotherapeutics into the brain tissue with curative intent for primary central nervous system lymphoma [26,27]. Because of the practical challenges in data collection during this invasive medical procedure, the number of datasets from each subject varies between one and four. Patient information as well as the number of measurements collected are shown in Table 1. Throughout the procedure, patients were administered a range of drugs, such as i.v. thiopental, benzodiazepine, atropine, and, i.a., mannitol for 30 s at 5 mL/s to open BBB. Anesthesia was induced and sustained with propofol, and the entire procedure typically lasted about 50 min. The data presented in this paper start with the mannitol infusion and after initial changes in BP and HR, slowly stabilize for a total period of 10 min. After the mannitol, no other vasoactive drugs were infused during the presented period.

### 2.1. Signal Collection and Processing

ECG and IBP signals were recorded using GE Datex-Ohmeda S/5 Compact (GE Healthcare, Helsinki, Finland), which is used for routine patient surveillance, with the sampling rate of Hz. A three-lead ECG signal was measured near the cardiac apex, in the para-sternal region. IBP was measured using an arterial catheter placed on the left arm. SCG signals were collected using an opto-mechanical sensor placed on the chest near the sternum, originally designed for use in MRI [28]. The sampling rate was 10 kHz.

Signal processing was performed using Matlab R2021a. After pre-processing with a 0.8 Hz high-pass Butterworth filter (order = 5), the R-peak detection in the ECG signal was conducted using the Pan–Tompkins algorithm. The maximum and feet of the IBP pulse were used to obtain systolic blood pressure (SBP) and diastolic blood pressure (DBP) values, from which mean arterial pressure (MAP) was later derived. Peaks in IBP signals were detected using a Matlab built-in function and verified by checking the distance to the corresponding R-peak, which were considered ground truth. SCG signals were bandpass-filtered using a 10th-order Butterworth filter with cut-off frequencies of 10 Hz and 35 Hz, in line with the recommendations for the sensor presented in [28], which corresponds to the SCG systolic spectrum in the dorsoventral axis [19,29,30]. The selected bandwidth ensures the removal of low-frequency baseline wandering because of respiration, as well as higher-frequency noise and artifacts associated with valve closure acoustics, while still preserving the key morphological features. SCG characteristic points were defined according to the existing literature, in which the maximum systolic peak corresponds to AO and the minimum before corresponds to IM [20,25,31], as shown in Figure 1. SCG pulse complexes were detected using a match filter, with the template pulse complex defined separately for each dataset. The final detection rate (see Table 2) was calculated in relation to all initially detected R-peaks in ECG signals. PEP_IM_ and PEP_AO_ were defined as time differences between ECG R-peak and characteristic points in SCG signals (see Figure 1).

### 2.2. Statistical Analysis

Acquired HR, BP, and PEP data were tested using the Kolmogorov–Smirnov test and assessed as normally distributed (*p* < 0.01). Values are presented as the mean ± standard deviation (SD). The detection rate was calculated as the number of detected SCG pulses divided by the reference number of detected ECG. Comparisons were tested using Student’s *t*-tests. Pearson’s correlation coefficient (R) was used to evaluate the beat-to-beat correlation between the PEP and BP. PEP-based BP estimation was made using 1st- and 2nd-degree regression models. The root mean square error (RMSE), coefficient of determination (R²), and Bland–Altman analysis were used for the evaluation of agreement between the SBP, DBP, and MAP estimations and their reference values. Bland–Altman rates included RPC, the reproducibility coefficient (±1.96* SD values), and CV, the coefficient of variation (SD of mean values in %).

## 3. Results

Eighteen datasets from nine patients were collected. From each dataset, we used a 10 min measurement period, starting with the infusion of mannitol. After the exclusion of parts affected by noise or artifacts, the mean detection rate in the analysis was estimated as 75%.

We checked the correlation of HR obtained using different devices. For HR based on the SCG signals (HR_SCG_), the correlation coefficient was 0.99 ± 0.01 (*p* < 0.001) when compared with HR calculated using RR intervals in ECG signals (HR_ECG_). The hemodynamic data of the study population, as well as calculated PEP_IM_ and PEP_AO_, are shown in Table 2.

**Figure 2:**
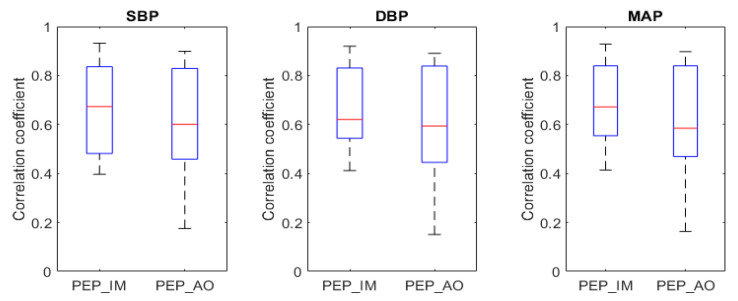
Distribution of Pearson’s correlation coefficient when PEP_IM_ and PEP_AO_ are compared with SBP, DBP, and MAP.

The beat-to-beat correlation of PEP_IM_ and PEP_AO_ was tested against values of SBP, DBP, and MAP (Figure 2). For all three pressures (SBP, DBP, and MAP), the correlation coefficients obtained for PEP_IM_ were higher than the ones obtained with PEP_AO_, with the differences being significant (*p* < 0.05). In the next step, PEP_IM_ values were used to create models for BP estimation. Four models were chosen for BP estimation: the commonly used linear model (#1), relatively understudied extended model with HR (#2) [32], and their second-degree versions (Model #3 and Model #4). Models’ equations are shown in Table 3. Parameters were derived using the least-squares method, individually for each dataset and separately for SBP, DBP, and MAP. Performance rates of both models are shown in Table 4.

**Figure 3:**
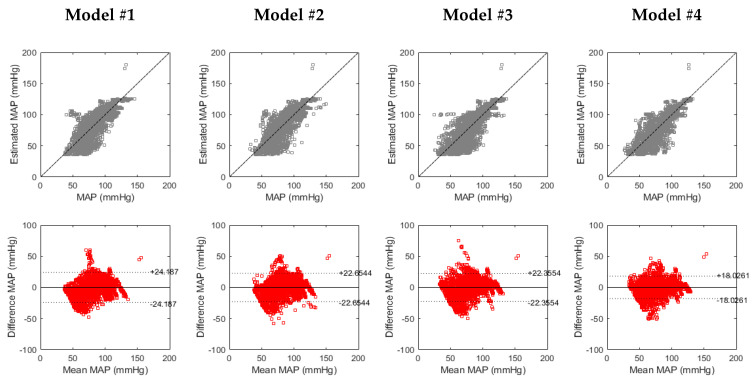
Results of correlation analysis and Bland–Altman plots comparing estimated MAP, based on PEP_IM_ values using four tested models, and reference MAP measured using IBP.

**Figure 4:**
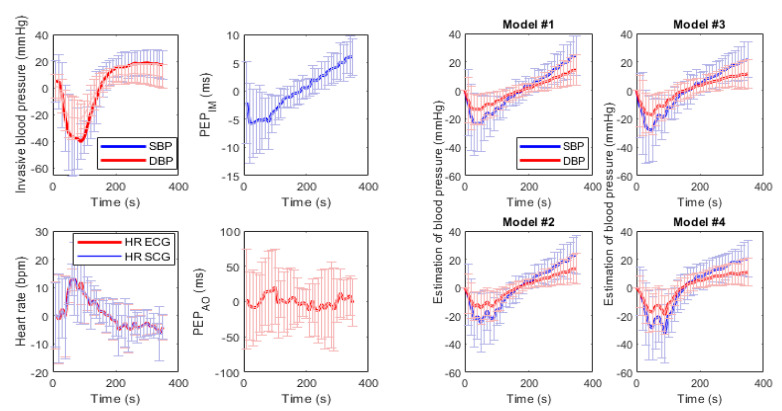
The average invasively measured blood pressure, heart rate, and pre-ejection period (PEP) changes during the period of the experiment, and the blood pressure estimation made using four models and PEP_IM_. All signals are unfiltered and normalized at baseline. The shaded areas indicate the standard deviation. The data represent 18 datasets from 9 subjects (see Table 1). Time = 0 is the time of mannitol infusion. SBP, systolic blood pressure; DBP, diastolic blood pressure. PEP_IM_: pre-ejection period measured to IM; PEP_AO_: pre-ejection period measured to AO.

Figure 3 presents the correlation and Bland–Altman analysis performed to compare PEP_IM_-based MAP estimation with four tested models, and MAP measured using IBP. The comparison was performed beat to beat, without BP values averaging in time segments. Figure 4 shows the dynamics of baseline deviations in SBP, DBP, HR, PEP_IM_, and PEP_AO_ as well as the estimated BP using four models.

**Figure 5:**
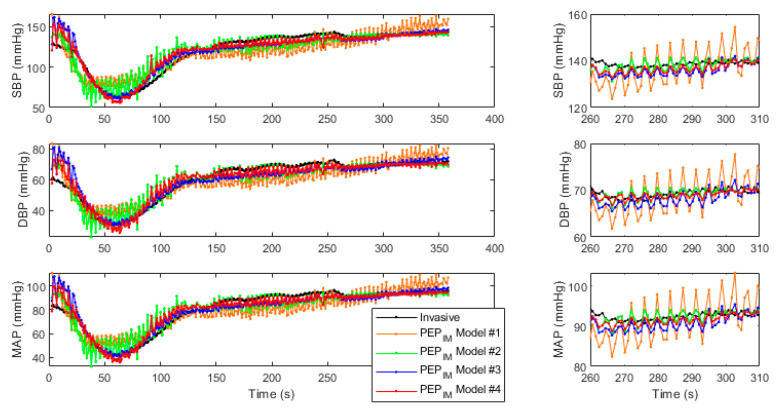
Representative plots of the beat-to-beat tracking of the SBP, DBP, and MAP based on invasive BP measurement and estimated BP using PEP_IM_-based models #1–#4.

Results achieved with models tested on PEP_IM_ values show lower levels of error when HR is added as a factor to the estimation. The best results can be observed with the second-degree extended equation used in Model #4 for DBP estimation (see Table 3). The RMSE in this case was equal to 6.8 ± 2.8 mmHg, as compared to estimating SBP with the same model, resulting in RMSE being 12.2 ± 4.9. On the other hand, using each model for SBP estimation resulted in RPC and RMSE values higher than in DBP and MAP estimation. The mean difference in all cases was equal to 0 mmHg. Bland–Altman plots reveal equal difference distribution for mean pressure values. Figure 4 shows normalized hemodynamic changes observed in IBP and HR, as well as SCG-based PEP values and BP changes estimated with four models and PEP_IM_ values. Figure 5 presents an example of beat-to-beat BP comparison between estimated and invasive SDP, DBP, and MAP values. Model #4 (red line) seems to best reflect the drop in BP after mannitol infusion. On the right side of the figure, the 50 s close ups of the graphs are shown. It can be noticed that the estimated values of BP reflect the beat-to-beat variance matching the respiratory ventilator rate of the subject.

**Figure 6:**
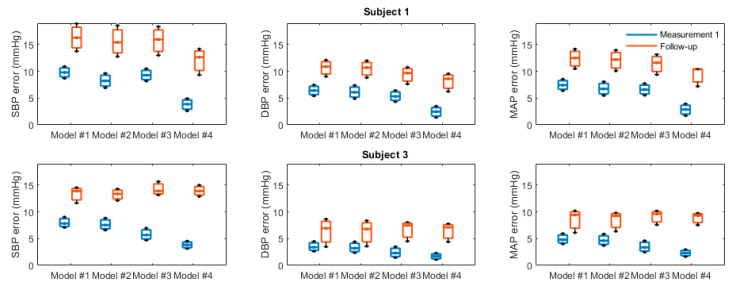
Performance comparison when models’ parameters generated for the first measurement are tested on three follow-up measurements without recalibration. The comparison was made for subjects who underwent four procedure repetitions, namely for Subject 1 and Subject 3 (see Table 1).

For subjects who underwent four procedure repetitions (Subjects 1 and 3), an additional step of intrasubject comparison was performed. Models’ coefficients were derived for the first measurement using regression, and then tested for BP estimation from PEP_IM_ in the three remaining measurements. The distribution of the mean absolute error of estimation is shown in Figure 6.

## 4. Discussion

### 4.1. Key Outcomes

We studied the potential of BP estimation based on the temporal relation between the ECG R-peak and SCG isovolumic moment (denoted as PEP_IM_). Four calibration models were used for BP modeling from PEP_IM_ values. Results were tested against simultaneously measured IBP. The findings suggest that investigating SCG signal shape fiducial points for BP estimation may be extending further than measurement of PEP_AO_, in particular when addressing other points than the aortic opening. When beat-to-beat values of PEP_IM_ and IBP are compared, a strong correlation can be observed. PEP_IM_ obtained higher mean correlation than PEP_AO_ when compared to both SBP and DBP. Notably, the SD of PEP_IM_ was only 7 ms with the corresponding MAP SD of 20 mmHg. It means that the changes in MAP cause very small changes in PEP_IM_ values. An SCG signal is measured directly above the heart, near the sternum, and the measured timings are not affected by the complex shape of the arterial tree. However, as a consequence, the method requires high accuracy in peak detection and a high sampling rate necessary to observe signal changes. When comparing the error resulting from different types of modeling, we observed that the performance rates for the second-degree models (Models #3 and #4) are slightly better than corresponding first-degree models (see Table 4). This complies with the theory that the relation between BP and PTT is nonlinear, which was suggested by researchers earlier [32,33,34]. It has been observed that nonlinear effects are particularly prominent when there is a significant change in BP values [34], which is in line with the results of the presented experiments. Of notable interest is the use of multivariate models extended with HR values (Models #2 and #4). The first-degree multivariate model shows mean RMSE values comparable to the second-degree polynomial model (Model #3). However, the second-degree multivariate model (Model #4) achieves significantly higher correlations and lower error levels compared to the other models tested. The quality and the overall accuracy of modeling might have been influenced by the detection rate (75% ± 24%, see Table 2). The undetected SCG pulses (and corresponding IBP values) were simply excluded from the further analysis, which potentially lead to less accurate predictions. It is especially concerning for measurement sets with detection rates <75%, as in the analyzed case, the distinctive change in BP was observed in a sudden spike during a specific part of the measurement, lasting approximately 100 s. Thus, the modeling would benefit from a higher detection rate, ensuring a more robust and precise prediction.

We assessed the accuracy of BP estimations using the Bland–Altman analysis and values of mean differences ± SD, which are recommended for the validation of new BP measurement devices [35]. According to the standards, a tolerable error of 10 mmHg or less (with an estimated probability of that error of at least 85%) is acceptable, taking into account the performance of currently available BP monitors. In our study, this requirement was met when estimating DBP and MAP using Model #4. Furthermore, in the intrasubject testing of the second-degree model extended with HR based on PEP_IM_ values, the mean error of MAP estimation in three follow-up measurements was in the range of 7.5 to 10.5 mmHg, without recalibration. Although the small sample size limits the ability to draw definitive conclusions, as the recommendation for validation studies is to use a sample size of at least 85 subjects [35], the results indicate a promising trend.

Data were collected in the supine position and under anesthesia. In the case of PTT measurement in the peripherals, there is a significant influence of body posture and limb position on the PTT levels [36,37]. Our hypothesis is that measuring only the subject’s torso leads to obtaining PEP_IM_ that is not affected by body posture. In our previous study conducted simultaneously with a Finapres device serving as a BP reference measurement device, the correlations of PEP_IM_ during sitting and supine positions were comparable (−0.67 and −0.68, respectively) [10].

It is important to emphasize that the aim of our work at this stage was showing a potential of using an SCG fiducial point other than AO for the purpose of BP estimation. While the experiments show relatively high correlations and low levels of errors, it is to be expected that this method alone is insufficient in creating a complete method for accurate BP estimation. Tested regression models were used to obtain the best estimation values, but the coefficients used in modeling cannot be generalized and hence are not presented at this stage. Further modeling will likely require supplementation by other parameters and/or deep learning techniques.

### 4.2. Strengths and Limitations

To the best of our knowledge, this is the first time that BP is estimated using the ECG R-peak and the corresponding SCG isovolumic moment; therefore, an SCG waveform can be defined as a substitute for a distal signal. In addition, IBP was used as a reference method, which is the most accurate method of continuous BP measurement. It is rare data to collect as a reference, due to its invasive nature, and thus particularly valuable for the evaluation of the new method performance. Furthermore, the presented method senses chest motions directly caused by heart activity, which potentially reflects central BP better than peripherally measured pulses. Finally, all analyses were performed beat to beat, without signal averaging.

The study has three limitations. The first one, and the most prominent, is the relatively small number of subjects. The study includes eighteen measurements collected from nine subjects on eighteen different days. Given the number of subjects, results can only be treated as preliminary and cannot be generalized. The second limitation is that data were collected during medical procedures, which required the patient’s anesthesia and use of vasoactive drugs, which might influence the relationship between BP and PEP_IM_. The rapid change in BP caused by the administration of mannitol enabled us to observe PEP_IM_ changes in the wide range of BP values (see Figure 4); however, such a setting is far from everyday scenarios.

### 4.3. Application Prospects and Future Improvements

SCG measurement in this study was performed using opto-mechanical accelerometers, which are MRI-compatible and were already used in numerous studies [9,28,38]. If the method will not be used in MRI environments, SCG can be realized using MEMS technology in a fairly simple and cost-effective way. Utilizing the presented method could provide a valuable and easy-to-use solution for long-term tracking of both HR and BP oscillations in a clinical setting, e.g., in an emergency room or in sleep studies. Using SCG also allows for breathing monitoring, as the chest motions caused by inhaling and exhaling are sensed by the SCG as well. Moreover, it is worth noting that there is potential for utilizing this solution as a wearable technique. Traditionally, PTT-based BP estimation requires the use of at least two separate sensing areas, which renders it more impractical for wearable applications. The presented method also makes it possible for the realization of a device that consists of several modalities in one measurement unit placed on the chest, as presented in [39].

Although SCG has been previously used to define a proximal timing in detecting the pulse transit time to the peripherals [40,41], a relatively understudied concept is to use the cardio-mechanical timing as an equivalent of a distal point and combining it with ECG measurement. The fact that both sensors would be placed on the chest could potentially result in values reflecting the central BP, which is the pressure in the aorta that can significantly differ from brachial (peripheral) pressure. Systolic BP (SBP) is amplified by a level that varies greatly from patient to patient: two individuals can have the same brachial cuff pressures, but their central pressures can differ by >30 mmHg [42,43,44]. As vital organs are exposed to central rather than brachial pressures, central BP seems to be more relevant for cardiovascular disease pathogenesis, as well as for reflecting end-organ damage of the heart, brain, and kidneys [45]. Furthermore, central BP can provide clinically useful information, aiding the prediction of future cardiovascular events, such as myocardial infarction or stroke; neurodegeneration [46]; and all-cause mortality [47]. There are devices available on the market, utilizing brachial cuffs and then estimating central BP values using generalized transfer functions with acceptable accuracy [48]. However, all measurements utilizing peripheral vascular function are easily affected by various confounding factors, such as temperature changes in arms, position and movements of arms, and the condition of peripheral circulation affected by age and different circumstances and peripheral vascular diseases [49]. Pulse sensing in a small distance might result in the detection method being prone to peripheral vessel properties.

This study is considered as an introduction of the method and its initial comparison in a clinical setting against gold-standard invasive BP measurement. It aims to draw attention to the potential of different SCG fiducial point characteristics, particularly in how they reflect BP values. Although at this point the ECG-PEP_IM_ delay does not meet the accuracy expected from a standalone method [35], it certainly has prospects to be included as a part of larger, multiparameter analyses, as in this study the quality result was obtained even at the beat-to-beat scale. Moreover, the presented study only observed relative values of BP oscillations. In order to obtain absolute BP values, calibration protocols need to be established, which were not included in this study.

Certain technical and processing improvements are planned. As recently pointed out in [50], the detection of SCG fiducial points based on reference ECG signals can result in detection errors. The potential misalignments and waveform distortions resulting from rescaling signals recorded using different modalities, equipment, and sampling rates could have influenced the detected fiducial point timings and, further, the results. In our detection algorithm, we apply certain proofing of peak detection in each beat, but we are also investigating the detection methods that do not rely on the reference signals. However, the advantage of our current standard signal processing techniques is that they are cost-effective and potentially suitable for wearable devices without demanding high computational power. Our future improvements focus on maximizing accuracy and automation, while being computationally efficient and suitable for a real-time analysis. In terms of hardware, an important step would be to incorporate both ECG and SCG in one device, in order to achieve synchronous acquisition. The setup might also benefit from using an additional accelerometer, placed separately and used solely for measurement of reference noise. This would potentially improve the detection rate and BP predictions, especially in a clinical setting, where sudden momentous changes in BP levels might appear.

Since the results obtained in our experiments are promising, extended follow-up studies across different populations, various health conditions, and measurements outside of a hospital are in place, in order to ensure the method’s effectiveness in diverse settings.

## 5. Conclusions

We observed that combining ECG and SCG measurements can provide different results depending on the chosen characteristic point in SCG. PEP_IM_ may provide a valuable indicator for beat-to-beat BP estimation. Based on the tested data, estimations based on the presented PEP_IM_ method provided high correlation levels and low errors when compared to invasively measured BP. These findings suggest that continuation studies including large populations of subjects are worth conducting, with the further incorporation of PEP_IM_ levels in multiparameter models.

## Figures and Tables

**Table 1:** Subject information.

Subject Number	Gender	Age	Number of Measurements
1	M	61	4
2	F	20	1
3	M	64	4
4	F	68	2
5	F	65	1
6	F	39	2
7	F	62	1
8	M	63	1
9	M	59	2
TOTAL	4M, 5F	56 ± 16	18

**Table 2:** Statistics of the hemodynamic data.

**Heart rate (bpm)**	74.9 ± 13.3
**SBP (mmHg)**	120.4 ± 27.4
**DBP (mmHg)**	69.2 ± 18.0
**MAP (mmHg)**	86.3 ± 20.4
**PEP_IM_ (ms)**	79.0 ± 7.0
**PEP_AO_ (ms)**	75.0 ± 12.1
**Detection rate (%)**	75 ± 24

**Table 3:** Conversion models used in the study.

**Model #1**	a_11_ × PEP_IM_ + c_1_
**Model #2**	a_21_ × PEP_IM_ + b_21_ × HR + c_3_
**Model #3**	a_32_ × PEP_IM_^2^ + a_31_ × PEP_IM_ + c_3_
**Model #4**	a_42_ × PEP_IM_^2^ + a_41_ × PEP_IM_ + b_42_ × HR^2^ + b_41_ × HR + c_3_

**Table 4:** Performance of SBP, DBP, and MAP estimation using different models.

		Model #1	Model #2	Model #3	Model #4
SBP	RMSE (mmHg)	16.5 ± 5.6	15.3 ± 6.0	15.3 ± 5.0	12.2 ± 4.9
	R	0.79	0.82	0.82	0.89
	R^2^	0.63	0.68	0.68	0.79
	RPC (mmHg)	33.81	31.72	31.51	25.71
	CV (%)	14.22	13.34	13.26	10.81
DBP	RMSE (mmHg)	9.4 ± 3.4	8.7 ± 3.5	8.6 ± 3.3	6.8 ± 2.8
	R	0.83	0.85	0.85	0.91
	R^2^	0.68	0.72	0.74	0.83
	RPC (mmHg)	19.86	18.6	18.23	14.6
	CV (%)	14.47	13.6	13.27	10.6
MAP	RMSE (mmHg)	11.7 ± 4.0	10.8 ± 4.2	10.8 ± 3.7	8.5 ± 3.4
	R	0.81	0.83	0.84	0.90
	R^2^	0.65	0.69	0.7	0.81
	RPC (mmHg)	24.18	22.65	22.36	18.02
	CV (%)	14.16	13.26	13.09	10.55

## Data Availability

The datasets presented in this article are not readily available due to privacy concerns. Access to these sensitive medical data is restricted to protect patient confidentiality. For any specific data requests, please contact the corresponding author.
